# Antioxidant and Stress Resistance Properties of Flavonoids from Chinese Sea Buckthorn Leaves from the Qinghai–Tibet Plateau

**DOI:** 10.3390/antiox13070763

**Published:** 2024-06-25

**Authors:** Jinmei Zhao, Yumei Jiang, Yang Bi, Juan Wei

**Affiliations:** College of Food Science and Engineering, Gansu Agricultural University, Lanzhou 730070, China; zhaojinmei01085@st.gsau.edu.cn (J.Z.); jym316@gsau.edu.cn (Y.J.)

**Keywords:** Chinese sea buckthorn, leaves, flavonoids, antioxidant activity, stress resistance

## Abstract

The unique ecological environment of the Qinghai–Tibetan Plateau has endowed Chinese sea buckthorn leaves with rich bioactivities. In this study, we investigated the bioactivity and stress resistance mechanisms of flavonoids derived from Chinese sea buckthorn leaves (FCL) native to the Qinghai–Tibet Plateau. Our analysis identified a total of 57 flavonoids, mainly flavonol glycosides, from FCL, of which 6 were novel flavonoids. Isorhamnetin glycosides, quercetin glycosides and kaempferol glycosides were the three most dominant classes of compounds in FCL. In particular, isorhamnetin-3-*O*-glucoside-7-*O*-rhamnoside emerged as the most abundant compound. Our results showed that FCL possesses potent antioxidant properties, as evidenced by its ability to effectively scavenge DPPH free radicals and demonstrate ferric reducing antioxidant power (FRAP) and oxygen radical absorbance capacity (ORAC) levels comparable to Trolox, a well-known antioxidant standard. Furthermore, FCL showed remarkable efficacy in reducing reactive oxygen species (ROS) levels and malondialdehyde (MDA) levels while enhancing the activities of key antioxidant enzymes, namely superoxide dismutase (SOD) and catalase (CAT), in *Caenorhabditis elegans*, a widely used model organism. Mechanistically, we elucidated that FCL exerts its stress resistance effects by modulating of transcription factors DAF-16 and HSF-1 within the insulin/insulin-like growth factor-1 signaling pathway (IIS). Activation of these transcription factors orchestrates the expression of downstream target genes including *sod-3*, *ctl-1*, *hsp16.2*, and *hsp12.6*, thus enhancing the organism’s ability to cope with stressors. Overall, our study highlights the rich reservoir of flavonoids in Chinese sea buckthorn leaves as promising candidates for natural medicines, due to their robust antioxidant properties and ability to enhance stress resistance.

## 1. Introduction

Chinese sea buckthorn (*Hippophae rhamnoides* subsp. *sinensis* Rousi) is an important subspecies of *Hippophae rhamnoides* L., which accounts for about 85% of all sea buckthorn in China. Chinese sea buckthorn, including wild forests and artificial forests, is a pioneering species for windbreak and sand fixation and has good economic value and ecological efficiency [1,2]. It is mainly distributed on the Loess Plateau, Inner Mongolia Plateau, and Qinghai–Tibet Plateau of China [2]. The Tibetan Plateau, which serves as the primary source of sea buckthorn in China, provides unique conditions for the secondary metabolism of sea buckthorn. These conditions result from its high altitude, low latitude, and intense ultraviolet radiation environment [1,3].

Flavonoids are the major active constituents of Chinese sea buckthorn leaves. They are mainly present in the form of flavonoid aglycones and flavonoid glycosides, which include flavones, flavonols, flavanols, flavanones, dihydroflavonol, chalcones, and procyanidins [4]. Studies by Wei et al. (2022) and Kang et al. (2019) have identified different flavonoids in sea buckthorn leaves [5,6]. Flavonols and their glycoside derivatives are particularly abundant and are formed by combining of flavonoid aglycones such as quercetin, isorhamnetin, and kaempferol with glycosyl groups such as glucose and rhamnose via glycosidic bonds [7,8]. Major flavonols in Chinese sea buckthorn leaves include isorhamnetin-3-glucoside, myricitrin, rutin, isorhamnetin-rhamnoside, kaempferol, quercetin, and hyperin [5,9]. Other flavonol derivatives such as isorhamnetin-3-glucoside-rhamnoside, narcissoside, nicotiflorin, rutin, and isoquercitrin have also been reported [2].

Sea buckthorn leaf extract exhibits potent antioxidant activity. Research indicates that the ethyl acetate extract of sea buckthorn leaves effectively scavenges superoxide anions (O_2_^−^·), showing a significant dose–response relationship [10]. Similarly, the methanol extract of sea buckthorn leaves demonstrates effective scavenging of DPPH free radicals, with an activity reaching 54.17 mg TE·g^−1^ [11]. Moreover, Chinese sea buckthorn leaf extract not only exhibits high cellular antioxidant capacity but also possesses anti-HepG2 cell proliferation properties [5]. Furthermore, it reduces oxidative stress in diabetic kidneys by decreasing the accumulation of advanced glycation end products (AGE), thereby ameliorating kidney damage [12]. It also shows cytoprotective and antioxidant properties against oxidative stress in mouse macrophages [13].

Despite previous characterizations of the flavonoid composition in Chinese sea buckthorn leaves, these studies predominantly used traditional solvent extraction and ultrasonic-assisted extraction methods, often resulting in incomplete identification of flavonoid types. Additionally, investigations into the antioxidant activity of flavonoids from Chinese sea buckthorn leaves sourced from the Tibetan Plateau, together with their anti-stress effects and underlying molecular mechanisms, remain largely unexplored. Therefore, this study aims to address these gaps by preparing a flavonoid extract (FCL) from Chinese sea buckthorn leaves using ultra-high pressure-assisted technology and AB-8 macroporous resin purification methods. Subsequently, the flavonoids in FCL will be systematically characterized using UPLC-ESI-QTOF-MS/MS and UPLC-PAD techniques. Moreover, the antioxidant activity of FCL will be evaluated to elucidate its anti-stress effects and mechanisms in a *C. elegans* model.

## 2. Material and Methods

### 2.1. Materials

Chinese sea buckthorn leaves were harvested from 10 different sampling sites in Lexiu Town, Hezuo City, Gannan Tibetan Autonomous Prefecture, Gansu Province, China in September 2021 (longitude 102.92′ E, local latitude 34.89′ N, about 3028 m above sea level), and the samples were collected by the S-shape sampling method, mixed, and then sampled according to the quartering method. After natural drying (28 ± 2 °C, RH 38 ± 2%), the leaves were crushed with a high-speed multifunctional pulverizer (H2489, Hebei Zhizhong Machinery Technology Co., Ltd., Xingtai, China) and sieved with a 40-mesh sieve, after which the sieved powder was treated with petroleum ether by Soxhlet extraction for 2 h. The sea buckthorn leaf powder was collected and placed at room temperature to evaporate the petroleum ether to dryness and then packed in sealed packages and refrigerated for further study.

We purchased *Escherichia coli* OP50 and wild-type N2 *Caenorhabditis elegans* from Fujian Shangyuan Biological Science & Technology Co., Ltd. (Fuzhou, China). TJ375, TJ356, CF1553, CL2166, and LD1 were purchased from the official website of Caenorhabditis Genetics Center (CGC). Without any specific instructions, *C. elegans* were maintained on agar plates at 20 °C using *E. coli* OP50 as the food source. Simultaneous treatment of adult *C. elegans* using a sodium perchlorate bleaching procedure to ensure that the growth period of each *C. elegans* remained constant during the experiment [14].

### 2.2. Preparation of Leaf Flavonoids

Based on our previous studies, the defatted sea buckthorn leaf powder was extracted with 50% ethanol by ultra-high pressure-assisted extraction under the conditions of pressure 143 MPa, temperature 44 °C, pressure hold time 3 min, and liquid–solid ratio 41:1. AB-8 macroporous resin was used to purify the crude extract, and the eluate was concentrated and lyophilized to obtain the purified sea buckthorn leaf flavonoids (FCL).

### 2.3. Identification of FCL Flavonoids

Identification and confirmation of chromatographic peaks by QTOF mass spectrometer (1290-6546, Agilent Technologies Co., Ltd., Santa Clara, CA, USA). Separation by chromatography was performed on a ZORBAX Eclipse Plus C18 column (3.0 × 100 mm, 1.8 μm). A binary gradient elution system consisting of water–formic acid (100:0.1, *v*/*v*) (A) and acetonitrile (B) was used as follows: 0–2.5 min, 14% B; 2.5–5 min, 14–18% B; 5–10 min, 18–19% B; 10–17.5 min, 19–28% B; 17.5–22.5 min, 28–32% B; 22.5–28.5 min, 32–45% B; 28.5–30 min, 45–98% B. The sample had an injection volume of 2 μL and a flow rate of 0.3 mL·min^−1^. The ionization source employed in this study was ESI, and the MS spectra were scanned with the scanning range set to *m*/*z* 100–1100 [9].

### 2.4. Quantification of the Major Flavonoid Components of FCL

The determination was made using ACQUITY UPLC (Waters, Milford, MA, USA), and all separations were carried out on a ZORBAX SB-C18 column (4.6 × 250 mm, 5 µm). Acetonitrile (B) and water–formic acid (100:0.1, *v*/*v*) (A) were utilized in a binary gradient elution system. 0–5 min, 86% A; 5–10 min, 86–82% A; 10–20 min, 82–81% A; 20–35 min, 81–72% A; 35–45 min, 72–68% A; 45–55 min, 68–55% A; 55–58 min, 55–86% A; 58–60 min, 86% A were the steps in the gradient elution process. Full-wavelength scanning was performed using the photodiode array detector (PAD), and 360 nm was ultimately chosen as the detection wavelength. The temperature of the column was kept at 30 °C, and the volume of injection was 10 μL, while the flow rate was 1 mL·min^−1^ [9].

### 2.5. Determination of Antioxidant Activity In Vitro

The DPPH free radical scavenging ability follows the method proposed by Zhang et al. (2010) [15]. FCL and Trolox reference standards were prepared in mixtures with 50% ethanol to create solutions ranging from 0–0.5 mg·mL^−1^. A 10 μL quantity of sample solution and 190 μL DPPH (50 mg·L^−1^) solution were added to ELISA plates. After reacting in dark for 30 min, the absorbance was measured at a wavelength of 517 nm. Results were expressed using DPPH free radical scavenging (%), with Trolox reference standards as positive controls.

The ferric reducing antioxidant power (FRAP) was determined using the kit. FCL was prepared as a 0.01–0.05 mg·mL^−1^ solution with 50% ethanol. The 950 μL of FRAP working solution and 50 μL of sample solution were mixed completely and the reaction was carried out for 20 min and then the absorbance was measured at 593 nm. FRAP values were expressed as mg Trolox (TE)·mL^−1^.

The oxygen radical absorbance capacity (ORAC) was based on the method proposed by Zhang et al. (2010) [15]. Briefly, 20 μL Trolox standard solution, 20 μL phosphate buffer (blank), and 20 μL sample solution were added to ELISA plates and incubated in a fluorescence microplate reader at 37 °C for 10 min. Then, 200 μL 6.0 μmol·L^−1^ fluorescein sodium working solution was added to ELISA plates, and after shaking incubation for 20 min, 20 μL 119 mmol·L^−1^ AAPH solution was added to each well. The fluorescence values of each well were measured every 5 min for a total of 31 measurements. The net area under the curve (AUC) was determined by subtracting the AUC of the blank from the AUC of the sample/Trolox fluorescence intensity. The ORAC values of the samples were computed by plotting the standard curve derived by the net AUC of Trolox; results were expressed in mmol TE·g^−1^ DW.

The method proposed by Adom and Liu (2005) was used as the basis for peroxide radical scavenging capacity (PSC) determination [16]. Briefly, 100 μL sample solution, 100 μL Trolox standard solution, and 100 μL phosphate buffer (blank) were added to the ELISA plate, and then 100 μL DCFH-DA solution and 80 μL 200 mmol·L^−1^ AAPH were added to each well, respectively. Fluorescence values were measured every 2 min at 37 °C and recorded for 40 min. Construct a standard curve based on the curve area of the Trolox dynamic curve to calculate the PSC value of the sample. The result is expressed as mmol TE·g^−1^ DW.

### 2.6. Determination of FCL Stress Resistance

#### 2.6.1. Heat and Oxidative Stress Assays

Synchronized L4-stage nematodes were selected and placed on NGM plates containing three different mass concentrations of FCL (50, 200, and 400 µg·mL^−1^ in *E. coli* OP50 bacterial solution, respectively), all of which contained 150 μM of 5-fluorouracil to inhibit the reproduction of nematode offspring. M9 buffer was used in place of FCL solution in the control group. Each group was set up with 3 NGM plates, and 40 nematodes were transferred to each plate and incubated at 20 °C. In the heat stress experiment, nematodes were treated with FCL for 96 h, and the culture temperature was changed from 20 °C to 35 °C, and then the culture was continued at constant temperature. Numbers of surviving nematodes in each group were monitored and counted at 2-h intervals until all nematodes died, for a total of three independent experiments. In the oxidative stress test, the nematodes were fed with FCL for 96 h and placed on NGM plates with 10 mM H_2_O_2_. The nematodes were observed and counted at 1 h intervals until all nematodes had died, for a total of three independent experiments [17].

#### 2.6.2. Determination of Reactive Oxygen Species (ROS) Levels

The nematodes were treated with FCL for 96 h, and then they were exposed to heat stress for 6 h at 35 °C to cause oxidative damage. In contrast, the control group did not receive either heat shock or FCL treatment. After three rounds of washing in M9 buffer, the nematodes on NGM plates were moved to an opaque 96-well plate that was filled with 50 µL of 100 µmol·L^−1^ DCFH-DA and 50 µL of M9 buffer solution. Following two hours of incubation at 20 °C, the nematodes were removed from the plate and placed on 2% agarose slides. Next, fluorescence intensity was measured with a fluorescence microscope [18]. Images under bright field and fluorescent field were recorded separately for each nematode and superimposed using Image Pro Plus 6.0 software.

#### 2.6.3. Determination of Malondialdehyde (MDA) Content and Superoxide Dismutase (SOD) and Catalase (CAT) Activities

The nematodes were treated with FCL at 20 °C for 96 h and then were exposed for 6 h to heat stress at 35 °C to induce oxidative damage. After three rounds of washing in M9 buffer, the nematodes on NGM plates were moved to centrifuge tubes, with approximately 800 nematodes per group. The nematodes were centrifuged at 5000 rpm·min^−1^ for 1 min, discarding the supernatant, and washing three times with M9 buffer solution. The nematodes were then crushed with a pestle provided in the kit, and the precipitate was adjusted with 1 mL of M9 buffer. Centrifuge the homogenate at 5000 rpm at 4 °C for 10 min and set the supernatant aside for testing. According to the instructions of the reagent kit (Soraibao Technology Co., Ltd., Beijing, China), MDA content and CAT, SOD activities were determined in the homogenate of nematodes, and the enzyme activities were expressed as U·mg pro^−1^.

#### 2.6.4. RNA Extraction and Determination of Gene Expression

Transfer of approximately 2000 synchronized nematodes to plates with or without 200 μg·mL^−1^ FCL was incubated at 20 °C for 48 h, then heat stressed at 35 °C for 6 h to induce oxidative damage. Total RNA was extracted using TRIzol reagent and reverse transcribed into cDNA using UnionScript First-stand cDNA Synthesis Mix for qPCR kit (Genesand Biotech Co., Ltd., Jinan, China). The transcript levels of *daf-2*, *akt-2*, gcs-1, *daf-16*, *gst-4*, *sod-3*, *hsf-1*, *hsp12.6*, *ctl-1*, *hsp16.2*, and *skn-1* genes were detected using GS AntiQ qPCR SYBR Green Fast Mix real-time fluorescence quantitative PCR kit (Genesand Biotech Co., Ltd.). The 2^−ΔΔCT^ method was used to assess relative gene expression levels and normalize them to the expression of the gene *β-actin*.

#### 2.6.5. Detection of Nuclear Translocation of DAF-16::GFP and SKN-1::GFP

LD1 (SKN-1::GFP) and TJ356 (DAF-16::GFP), synchronized transgenic strains of the L4 stage, were placed on NGM plates and treated at 20 °C for 48 h with or without 200 µg·mL^−1^ FCL, and groups of nematodes were divided into two parts: one part was treated with heat stress for 6 h at 35 °C, and the other was untreated. Nematodes were washed with M9 buffer solution and transferred to 2% agarose slides for observation under a fluorescence microscope. DAF-16 localization in each nematode was divided into 3 types (nucleus, cytoplasm, and intermediates), taking into account the primary location of DAF-16::GFP [19], with a count of at least 60 nematodes per group.

#### 2.6.6. Determination of GFP Fluorescence Intensity in Transgenic Strains

Synchronized transgenic nematodes TJ375 (HSP-16.2::GFP), CF1553 (SOD-3::GFP) and CL2166 (GST-4::GFP), and were cultured for 48 h in NGM plates with or without 200 µg·mL^−1^ FCL. After heat stress treatment for 6 h at 35 °C, nematodes were washed with M9 buffer solution and transferred to 2% agarose slides for observation under a fluorescence microscope.

### 2.7. Data Statistics

All tests were repeated three times, and data are presented as mean ± standard deviation. All data were analyzed by analysis of variance (ANOVA) with Duncan’s test of significance of differences using SPSS 20.0 statistical software. ChemDraw20.0 was used to draw the chemical structural formula of flavonoids, and Origin 9.0 was used to draw the graph. To determine the lifespan of *C. elegans*, Kaplan–Meier survival analysis was performed and plotted using Graphpad Pism9.5. The fluorescence images of *C. elegans* were processed and analyzed using Image Pro Plus 6.0 software, and the relative fluorescence values were statistically analyzed, with at least 15 images in each group.

## 3. Results

### 3.1. Identification of Flavonoids in FCL

A total of 60 flavonoids were found in FCL (Supplementary Figure S1), of which 57 were finally identified. Although there were characteristic fragments of flavonoid aglycones present in the MS/MS fragments of compounds **37**, **54** and **57**, they were not identified due to insufficient structural information (Table 1).

**Figure 1:**
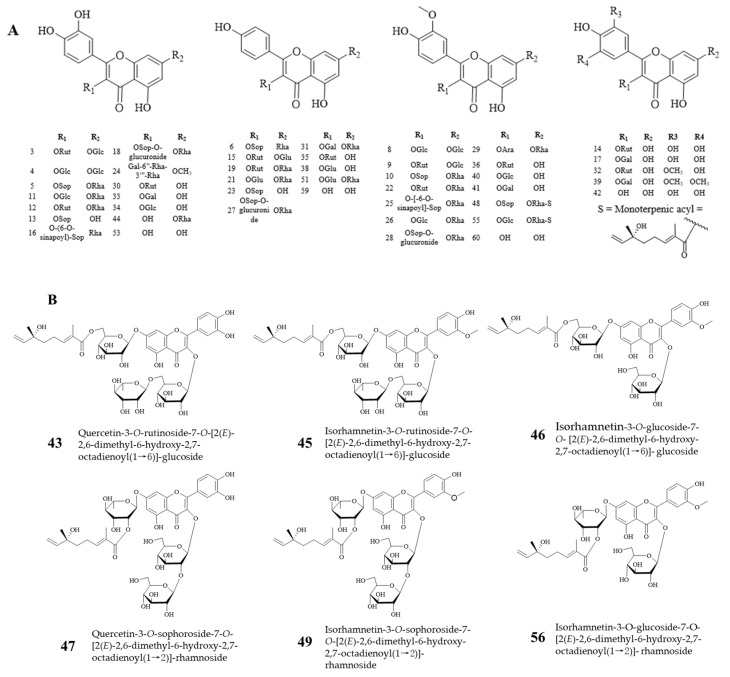
Flavonols and 6 new flavonoids in Chinese sea buckthorn leaves. (**A**) Flavonols, (**B**) 6 new flavonoids.

According to the MS/MS fragment information of the flavonoid aglycone reference standard (Supplementary Figure S2), it can be inferred that the major flavonoid components of FCL are flavonol glycosides derived from isorhamnosin, quercetin, kaempferol, and myricetin. In MS/MS spectrometry, glycosidic bond cleavage is a characteristic cleavage mode of flavonoid glycosides [9,20]. As an example, compound **9** had an [M-H]^−^ ion at *m*/*z* 785.2126, and its molecular formula was speculated to be C_34_H_42_O_21_. The four major fragment ions were observed at *m*/*z* 623.16, 477.10, 315.05, and 300.03. The fragment ion at *m*/*z* 623.16 was raised as a result of the loss of glucose, and the loss of glucose further produced the ion at *m*/*z* 477.10. The fragment ion *m*/*z* 315.05 was generated by the loss of glucose at *m*/*z* 477.10, which was further cleaved to produce the same characteristic ion peaks 300.02 and 151.01 as the reference standard of isorhamnetin. Combined with Chemspider, Massbank information and MS/MS spectral data preliminary identification of compound **9** as isorhamnetin-3-*O*-rutin-7-*O*-glucoside, and its possible cleavage pathway was shown in Supplementary Figure S3. Based on the above fragmentation pattern, a total of 45 flavonols were identified from FCL (Figure 1) [20,21]. The 8 compounds **43**, **45**–**49**, **55** and **56** all contain an ionic fragment at *m*/*z* 166.0978, of which compounds **48** and **55** are identified according to the reference as 3-*O*-β-D-sophorosyl-kaempferol-7-*O*-{3-*O*-[2(*E*)-2,6-dimethyl-6-hydroxy-2,7-octadienoyl]}-α-L-rhamnoside and 3-*O*-β-D-glucosyl-kaempferol-7-*O*-{2-*O*-[2(*E*)-2,6-dimethyl-6-hydroxy-2,7-octadienoyl]}-α-L-rhamnoside [22], and deduced *m*/*z* 166.0978 as [2(*E*)-2,6-dimethyl-6-hydroxy-2,7-octadienoyl]^−^. Based on the ion fragmentation information, we identified the remaining 6 compounds. Take compound **47** as an example: its quasimolecular ion was *m*/*z* 937.2957 [M-H]^−^ with molecular formula C_43_H_54_O_23_, and five major ion fragments were observed at *m*/*z* 775.24, 609.14, 463.09, 447.08, and 301.03. The attributions of the fragment ions are: *m*/*z* 775.24 for [M-H-Glc]^−^, *m*/*z* 609.14 for [M-H-Glc-S]^−^, *m*/*z* 447.08 for [M-H-Glc-S-Glc]^−^, *m*/*z* 463.08 for [M-H-Glc-S-Rha]^−^, and *m*/*z* 315.05 for [M-H-Glc-S-Rha-Glc]^−^, from which compound **47** was presumed to be Quercetin-3-*O*-sophoroside-7-*O*-[2(*E*)-2,6-dimethyl-6-hydroxy-2,7-octadienoyl(1→2)]-rhamnoside, whose possible cleavage pathway is shown in Figure S4. In addition, three flavanols (compounds **1**, **2**, and **7**) and one flavane (**58**) were identified. Compounds **1** and **2** (*m*/*z* 305.0667) were speculated to be isomers based on their similar fragment paths, and fragment information was compared with a reference and identified as gallocatechin and epigallocatechin [23]. Based on Chemspider and Massbank databases, compound **7** (*m*/*z* 289.0715) was characterized as epicatechin, while compound **58** (*m*/*z* 271.0606) was identified as naringenin by comparison of MS and fragmentation information with the reference standard of naringenin.

The flavonoid components of FCL were mainly flavonol glycosides formed by the combination of flavonoid aglycones and glycosyl groups. The glycoconjugates mainly include glucose, rhamnose, rutinose, sophorose, arabinose and galactose. According to the glycosylation method, the flavonoid components of FCL are mainly classified as *O*-glycosides, and the glycosidic linkages were mainly formed at the 3-position of the C-ring and the 7-position of the A-ring (Figure 1). Among the 57 identified flavonol glycosides, there were 18 types of isorhamnetin glycosides, 16 types of quercetin glycosides, and 14 types of kaempferol glycosides, among which 6 compounds were new flavonoid compounds (**43**, **45**, **46**, **47**, **49**, and **56**), and 7 compounds (compounds **16**, **24**, **25**, **32**, **39**, **48**, and **55**) were detected for the first time in sea buckthorn leaves. The 3 unidentified flavonoids (compounds **37**, **54**, and **57**) were found for the first time in sea buckthorn.

### 3.2. Quantification of Major Flavonoids

FCL contained abundant flavonoids (64.35 ± 0.79 mg·g^−1^ DW) (Supplementary Table S1, Figure S5), more than olive leaves (4.92–18.29 mg·g^−1^ DW) and mulberry leaves (31.212 mg·g^−1^ DW) [24,25]. The flavonoid fraction of FCL could be classified into three classes according to the difference in the number of glycosidic chains, namely flavonoid disaccharide chain glycosides, flavonoid monosaccharide chain glycoside, and flavonoid aglycone. Among them, flavonoid disaccharide chain glycosides were the dominant component, followed by flavonoid monosaccharide chain glycoside, with flavonoid aglycone content accounting for only 13.78% of the total content. It supports the findings of Liu et al. [4] and suggests that the flavonoids abundant in Chinese sea buckthorn leaves were primarily flavonoid glycosides [19]. Its flavonoid glycosides were mainly derivatives of kaempferol, isorhamnetin, and quercetin. Among them, quercetin glycosides and isorhamnetin glycosides accounted for the largest portion (78.13%) of the total flavonoids, and their species were more diverse, suggesting that sea buckthorn leaves are a good source of isorhamnetin and quercetin derivatives. Compared to sea buckthorn berry flavonol profiles [8], kaempferol glycosides (totaling 17.39% of total flavonoids) were more abundant in sea buckthorn leaves. Among the 16 flavonoids quantified, the most abundant was isorhamnetin-3-*O*-glucoside-7-*O*-rhamnoside (17.74% of the total flavonoids), followed by quercetin-3-*O*-rutinoside (13.17%), kaempferol-3-*O*-sophoroside-7-*O*-rhamnoside (13.38%) and quercetin-3-*O*-galactoside (12.74%).

### 3.3. Antioxidant Activity of FCL In Vitro

**Figure 2:**
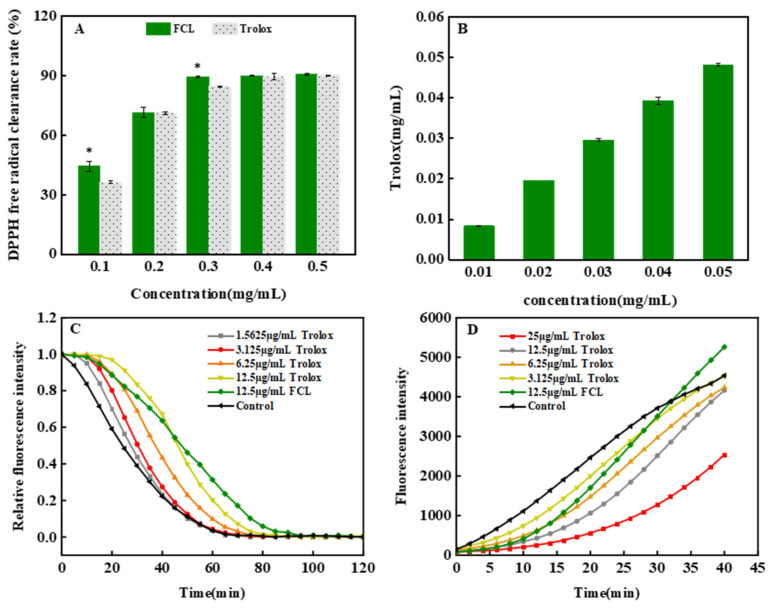
Effects of FCL on DPPH free radical scavenging ability (**A**), FRAP (**B**), ORAC (**C**), and PSC (**D**). Compared with the Trolox group, * indicates significant difference (*p <* 0.05).

A dose-dependent DPPH radical scavenging ability was observed for both FCL and Trolox (Figure 2A), with IC_50_ values of 0.123 mg·mL^−1^ and 0.157 mg·mL^−1^, respectively, suggesting that FCL scavenges DPPH radicals 1.28 times more than Trolox. The FRAP of FCL increased with the increasing concentration (Figure 2B), and in the concentration range of 0.01–0.05 mg·mL^−1^, the iron-reducing antioxidant power of FCL was equivalent to that of Trolox. Figure 2C, D plot the temporal kinetic curves of the relative fluorescence intensity of Trolox standard solution and FCL decaying and increasing with time. The ORAC value of FCL was 3.74 ± 0.22 mmol TE·g^−1^, which was 0.935 times higher than that of Trolox. The fluorescence intensity decay trend of 12.5 μg·mL^−1^ FCL was similar to that of Trolox (Figure 2C). Trolox and FCL showed a clear dynamic trend of fluorescence increase, and the fluorescence intensity increase trend of 25 µg·mL^−1^ FCL and 6.25 µg·mL^−1^ Trolox was similar (Figure 2D). Taken together, this indicated that FCL can effectively scavenge DPPH radicals and has iron-reducing antioxidant power and ORAC comparable to that of Trolox.

The radical scavenging reaction of DPPH and the FRAP reaction belong to the single electron transfer reaction, which mainly reflects the reducing ability to high valence ions [26] The antioxidant reaction of ORAC and PSC belongs to the hydrogen atom transfer mechanism, which mainly reflects the ability of substrates and antioxidants to compete for peroxy radicals [15]. Flavonoids can provide hydrogen atoms to free radicals, acting as good electron donors to inhibit the development of peroxide chain reactions [15,27]. Thus, we could infer that the strong antioxidant capacity of FCL is mainly due to its abundant flavonoid components.

### 3.4. The Effect of FCL on the Stress Resistance in C. elegans

#### 3.4.1. Effects of FCL Treatment on the Ability of *C. elegans* to Resist Oxidative and Heat Stress

**Figure 3:**
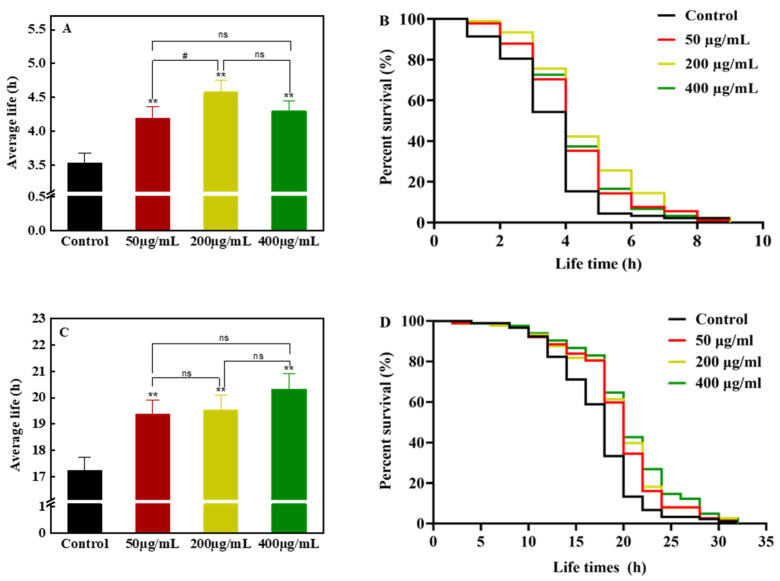
Effects of FCL on average lifespan (**A**), life curve (**B**) under oxidative stress, average lifespan (**C**) and life curve (**D**) under heat stress in *C. elegans*. The symbol ** indicates an extremely significant difference compared to the control group (*p* < 0.01), the symbol # indicates the comparison between different treatment groups (# *p* < 0.05), and ns indicates no significant difference.

Under oxidative stress treatment, the mean survival time of nematodes groups fed with three different concentrations of FCL was significantly higher than that of the control group (Figure 3A). The mean survival time of nematodes in the 200 μg·mL^−1^ FCL group was the longest and 29.58% higher than that of the control group. The survival curves of the FCL-treated group were generally shifted to the right compared with the control group (Figure 3B), suggesting that FCL could effectively enhance the tolerance to oxidative stress in nematode. High temperature can lead to metabolic disorders and enzyme inactivation in the body, generating large amounts of ROS and causing oxidative stress [28]. Under the heat stress treatment, the mean survival time of nematodes in all three FCL groups with different concentrations was extremely significantly higher than that of the control group (*p* < 0.01) (Figure 3C). Among them, the 400 μg·mL^−1^ FCL-treated group showed the longest survival time of nematodes. Survival curves were generally shifted to the right in the FCL-treated group compared with the control group (Figure 3D), indicating that FCL could effectively enhance the heat stress resistance of nematodes and showed strong antioxidant capacity. Since flavonoids such as quercetin and rutin could enhance the resistance of *C. elegans* to heat stress [29], and isorhamnetin and its derivatives had potential protective effects against oxidative stress in human RPE cells [30], it was suggested that the abundant isorhamnetin and quercetin derivatives in FCL may be mainly responsible for the enhanced resistance of *C. elegans* under stress conditions.

#### 3.4.2. Effects of FCL Treatment on ROS, MDA Levels, and SOD and CAT Activities

**Figure 4:**
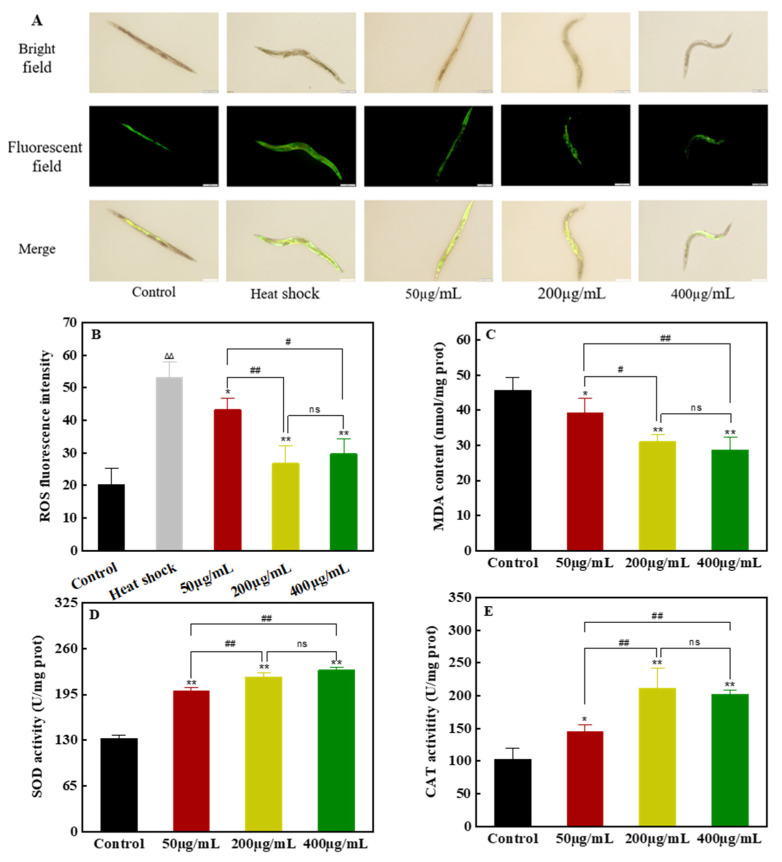
Effects of FCL on the levels of ROS and MDA and the activities of SOD and CAT in *C. elegans*. (**A**) Comparison of the fluorescence levels of ROS (scale 200 μm). (**B**) Quantitative determination of ROS fluorescence intensity. The symbol ∆∆ indicates that the difference was highly significant compared with the control group (*p* < 0.01), the symbol * indicates the comparison with the heat stress group (* *p* < 0.05, ** *p* < 0.01), and the symbol # indicates the comparison between different treatment groups (# *p* < 0.05, ## *p* < 0.01), and ns indicates no significant difference. (**C**) MDA content; (**D**) SOD activity; (**E**) CAT activity.

The ROS level in the heat stress-treated model group increased sharply (Figure 4A,B), which was 2.64 times that of the control group (without heat stress treatment), indicating that stress treatment could significantly increase the ROS level in *C. elegans*. Under heat stress, FCL treatment significantly reduced ROS levels, 50 µg·mL^−1^ FCL reduced ROS levels by 18.76%, and at high concentrations of 200 µg·mL^−1^ and 400 µg·mL^−1^, ROS levels in *C. elegans* were reduced by 49.62% and 44.08%, respectively. This indicates that FCL treatment could effectively remove ROS levels in *C. elegans*, with the high concentration of FCL being particularly effective in ROS removal. Similarly, the FCL-treated group resulted in *C. elegans* with lower MDA levels (Figure 4C), which were reduced by 14.22%, 32.0%, and 37.07% in the 50 µg·mL^−1^, 200 µg·mL^−1^ and 400 µg·mL^−1^ treatment groups, respectively, compared with the control. Peroxidation and oxidative stress in the organism are directly caused by excessive ROS [31]. The level of lipid peroxidation in the body can be indicated by the amount of MDA present, which is created by oxygen free radicals in cell membranes and the peroxidation of unsaturated fatty acids [30]. The findings of this study demonstrated that the FCL significantly reduced the amount of ROS and lipid peroxidation in nematodes under acute stress. Consequently, we deduced that FCL would improve oxidative stress resistance of *C. elegans* by inhibiting the accumulation of excess ROS and the onset of lipid peroxidation.

The SOD activity of nematodes fed with different concentrations of FCL were significantly higher than the control (*p <* 0.01). The SOD activities of the 50 μg·mL^−1^, 200 μg·mL^−1^, and 400 μg·mL^−1^ FCL groups were 50.92%, 65.86%, and 72.80% higher than those of the control group, respectively (Figure 4D). In the same way, *C. elegans* treated by various FCL doses exhibited significantly increased CAT activity compared to the control group (*p <* 0.05). The 200 μg·mL^−1^ and 400 μg·mL^−1^ FCL groups were 106.08% and 96.86% higher than the control group, respectively. This indicated that, under situations of heat stress, FCL might considerably increase the SOD and CAT activities and eliminate excess ROS, extending the survival rate of *C. elegans*.

### 3.5. The Molecular Mechanism of FCL Regulation of Stress Resistance in C. elegans

The insulin/insulin-like growth factor-1 signaling pathway (IIS) is a classical and conserved signaling pathway for aging regulation, which can regulate the activities of three transcription factors (DAF-16, SKN-1, and HSF-1), thereby inducing the expression of a series of genes related to stress response, homeostatic regulation, and metabolism [32]. To investigate if the IIS pathway has a role in FCL-mediated stress resistance in nematodes, we detected the expression levels of genes and proteins related to the IIS pathway.

**Figure 5:**
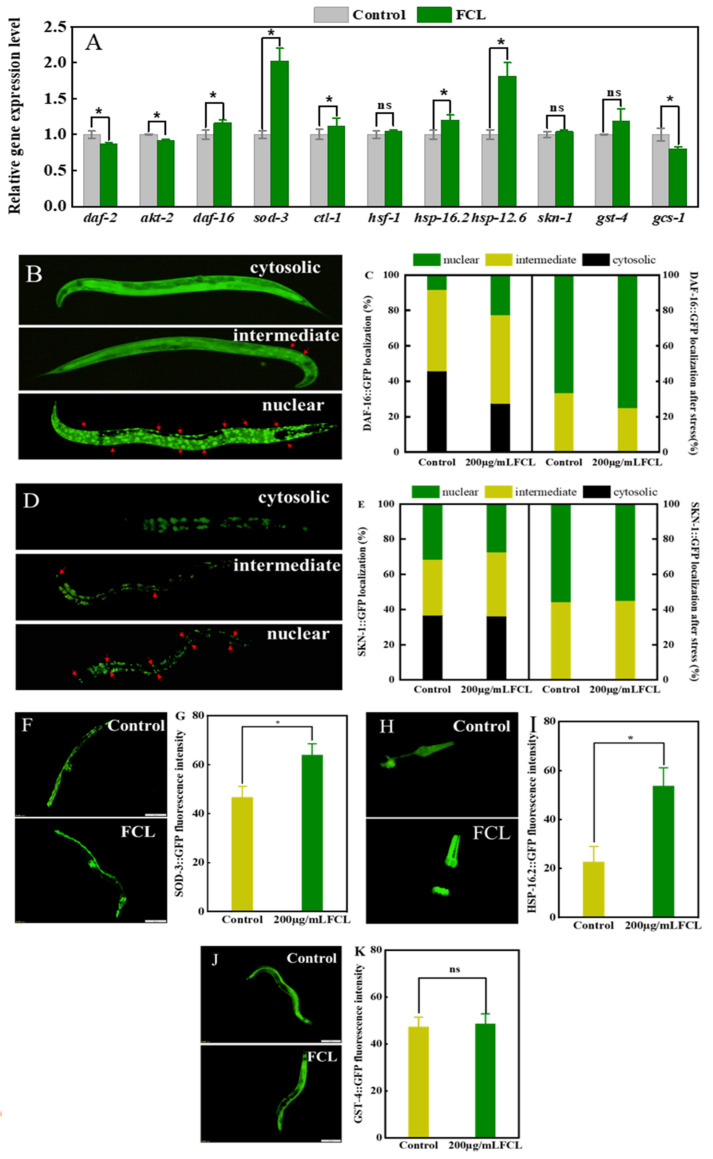
Effect of FCL on stress resistance-related genes and protein expression in *C. elegans*. (**A**) The expression level of stress resistance related genes. (**B**) Three kinds of DAF-16::GFP localization. (**C**) Histogram of the distribution ratio of DAF-16::GFP in *C. elegans*. (**D**) Three types of SKN-1::GFP localization. (**E**) Histogram of the distribution ratio of SKN-1::GFP in *C. elegans* (Red arrows indicate nuclear translocation). (**F**,**G**) SOD-3: GFP fluorescence expression of CF1553 strain. (**H**,**I**) HSP-16.2: GFP fluorescence expression of TJ375 strain. (**J**,**K**) GST-4::GFP fluorescence expression of CL2166 strain (scale 200 μm); * was significantly different (*p* < 0.05), and ns was not significantly different.

The 200 μg·mL^−1^ FCL treatment significantly decreased the transcript levels of *daf-2* and *akt-2* in the IIS pathway compared with the control (*p* < 0.05) and significantly increased the transcript levels of *daf-16* and its downstream genes, *ctl-1* and *sod-3* (*p* < 0.05), with a 1.02-fold increase in the expression of the *sod-3* gene (Figure 5A). In the DAF-16 nuclear translocation assay (Figure 5B), FCL treatment decreased the DAF-16::GFP expression in the cytoplasm and increased the proportion of *C. elegans* with its localization in the nucleus. In the control group, the proportion of DAF-16::GFP in the nucleus was 8.3%, while in the FCL-treated group, it increased to 22.5%; after the stress treatment, FCL increased the nuclear localization of DAF-16::GFP to 75.0% compared to 67.9% in the control group (Figure 5C). DAF-16 is an important transcription factor that influences the ability of *C. elegans* to resist stress [33]. The genes upstream of the IIS pathway, *daf-2* and *akt-2* are repressed in a stress environment, which prompts DAF-16 to dephosphorylate and translocate from the cytoplasm to the nucleus. There, it binds to the DNA promoter-binding region, inducing the expression of target genes and increasing the resistance of *C. elegans* to stress [30]. Results of this study indicated that FCL activated the transcription factor DAF-16, promoted its nuclear translocation, up-regulated the expression of the downstream target genes *sod-3* (sod gene) and *ctl-1* (CAT gene), and enhanced the resistance of *C. elegans*. This result was also verified in the CF1553 transgenic strain, in which FCL treatment dramatically up-regulated the SOD-3 protein expression level (Figure 5F,G).

FCL treatment increased gene expression of *hsf-1* compared to control, and its downstream genes *hsp-16.2* (small heat shock protein gene) and *hsp-12.6* increased by 20% and 81%, respectively (Figure 5A). Expression of HSP-16.2::GFP protein was significantly up-regulated by FCL treatment in the TJ375 transgenic strain (HSP-16.2::GFP) (Figure 5H,I). HSF-1 is a heat shock transcription factor that maintains protein homeostasis by regulating the expression of chaperone heat shock proteins, which can protect proteins in nematodes from damage caused by external stresses environmental stress [34]. The high expression of HSF-1-regulated HSP-16.2 improves cellular heat resistance and protein homeostasis in unknown organisms by preventing misfolding and translation [35]. The results indicated that HSF-1 and its downstream genes were significantly activated and had an important role in the enhancement of heat stress resistance activity of *C. elegans* by FCL.

No significant difference was observed in the expression level of *skn-1* compared to the control group. *Gst-4* expression was up-regulated but not statistically significant (*p >* 0.05); *gcs-1* expression was down-regulated (Figure 5A). The same results were found in the SKN-1 nuclear translocation assay, where the FCL-treated group had no significant effect on the level of nuclear translocation of SKN-1::GFP compared with the control group (Figure 5D,E). In addition, the expression of GST-4::GFP protein in the CL2166 (GST-4::GFP) transgenic strain was not significantly altered by FCL treatment (Figure 5J,K). *Skn-1*, a gene homologous to mammalian Nrf2, can augment the resistance to oxidative stress in *C. elegans* by modulating phase II detoxification genes [36]. However, the results showed that FCL treatment did not significantly affect *skn-1* gene and protein expression, which suggested that *skn-1* may not be related to the enhancement of *C. elegans* antioxidant capacity by FCL.

## 4. Discussion

Oxidative stress is caused by an imbalance between the generation of oxidants and the elimination of free radicals by antioxidants, and this imbalance leads to the destruction of biomolecules and cells, which is potentially destructive to the whole organism [12,28]. Therefore, enhancing the antioxidant capacity of the organism is an effective approach to defend against oxidative stress. The antioxidant defense system of organisms mainly includes endogenous antioxidant enzymes, endogenous non-enzymatic antioxidants, and exogenous antioxidants [29]. Some natural and harmless compounds, such as polyphenolics and flavonoids, are good exogenous antioxidants, which could effectively scavenge free radicals and reduce the damage caused by oxidative stress [14].

In this study, we used UPLC-ESI-QTOF-MS/MS and UPLC-PAD techniques to separate and characterize flavonoids with different polarities using binary gradient elution consisting of water–formic acid and acetonitrile [9], and found that FCL was rich in flavonoids. In terms of antioxidant activity, FCL could effectively scavenge DPPH radicals and possessed ferric reducing antioxidant power and oxygen radical absorption capacities comparable to those of Trolox, in which the ORAC value was 3.74 ± 0.22 mmol TE·g^−1^, higher than that of the forsythia flavonoids extract (0.928 mmol TE·g^−1^) and hawthorn extracts (1.17 mmol TE·g^−1^) [37,38], demonstrating strong in vitro antioxidant activity. Using *C. elegans* as a model, we induced oxidative stress with H_2_O_2_ and high temperatures (35 °C), which significantly increased ROS levels in nematodes, while FCL-fed nematodes had lower levels of ROS and resulted in a significant increase in their lifespan. This indicated that FCL could enhance the ability of stress resistance in nematodes through directly scavenging ROS. This effect was similar to that of some reported flavonoid compounds, which could also improve the oxidative stress resistance of nematodes, such as flavonol glycoside complanatoside A, Rhodiola rosea extract, quercetin, rutin, etc. [27,29,34].

In addition to exogenous antioxidants, endogenous antioxidant enzymes also play important roles in the cellular antioxidant defense system, such as SOD and CAT. SOD could disproportionate O_2_^−^· to H_2_O_2_ and O_2_, and CAT could catalyze H_2_O_2_ to produce water and O_2_ [39,40]. To verify if the stress resistance ability of FCL is associated with the antioxidant enzyme system, we evaluated antioxidant enzyme activities in nematodes and found that FCL treatment resulted in a significant increase in enzyme activities in nematodes. A number of studies have shown that the regulation of antioxidant enzyme gene expression greatly influences antioxidant enzyme activity, implying that FCL may enhance the defense mechanism of nematodes by modulating endogenous metabolic pathways.

The IIS pathway is a key regulatory mechanism for the growth, development, immune defense, and stress resistance of organisms [36,41]. The initiation of its kinase cascade is dependent on the phosphorylation of DAF-2 (insulin receptor), which then regulates the downstream signaling molecule AGE-1/PI3K (phosphatidylinositol kinase), followed by the further activation of serine/threonine protein kinases, including AKT, which in turn regulates downstream transcription factors [42]. DAF-16, SKN-1, and HSF-1 are three important transcription factors in the IIS pathway. The IIS kinase cascade regulates the nuclear translocation of DAF-16 and SKN-1, which can activate the downstream antioxidant enzyme (SOD, CAT, GST-4, GCS-1) genes and enhance the antioxidant capacity of the organism [33,34]. HSF-1 plays an important role in maintaining protein homeostasis by regulating the expression of heat shock proteins (HSP12.6, HSP16.2) [34]. The research results indicate that FCL treatment decreased *daf-2* and *akt-2* gene relative expression, which negatively regulates lifespan in the IIS pathway of nematodes, and increased the relative expression levels of *daf-16* and its downstream target genes, *sod-3*, *ctl-1*, and *hsp-16.2* in this pathway. At the same time, it promoted DAF-16 entry into the nucleus and enhanced the expression of DAF-16 and HSF-1-regulated downstream proteins SOD-3 and HSP-16.2, indicating that FCL could mediate stress resistance in nematodes through the IIS signaling pathway, which required the involvement of transcription factors DAF-16 and HSF-1.

**Figure 6:**
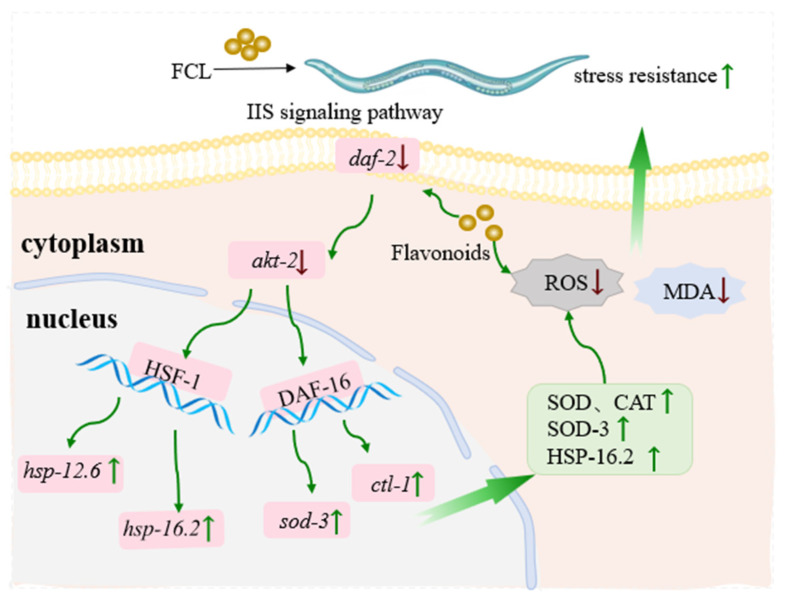
Pattern diagram of FCL regulation of stress resistance in *C. elegans*. The symbol ↓ indicates down-regulation of gene expression, the symbol ↑ indicates up-regulation.

Taken together, FCL, as an antioxidant, could directly scavenge free radicals to reduce ROS levels and achieve stress resistance and also modulate the IIS signaling pathway, activate the downstream transcription factors DAF-16 and HSF-1, and regulate the expression of antioxidant enzymes and heat shock proteins, to achieve the virtuous circle of alleviating oxidative stress and prolonging lifespan (Figure 6). To further explore the potential contribution of FCL’s antioxidant effects and its underlying mechanisms, we will investigate the signature flavonoids and conduct extra experiments, such as protein-specific analyses and more relevant mutational analyses.

## 5. Conclusions

In FCL, 57 flavonoids were identified, including 53 flavonols, 3 flavanols, and 1 flavan, with 6 new flavonoid compounds being detected in sea buckthorn leaves. In particular, flavonols, especially isorhamnetin-3-*O*-glucoside-7-*O*-rhamnoside, were predominant. FCL exhibited Trolox-like antioxidant activity and showed potential to enhance nematode stress resistance by modulating ROS and MDA levels, enhancing SOD and CAT activities, and activating antioxidant enzymes and heat shock protein genes in the IIS pathway. These findings highlight the antioxidant and stress resistance properties of Chinese sea buckthorn leaf flavonoids and provide insights for their application in functional foods.

## Figures and Tables

**Table 1:** Flavonoids in the leaves of Chinese sea buckthorn.

Compounds	Rt/min	Formula	[M-H]^−^ *m*/*z*	Fragment Ion *m*/*z*	Identification
**Flavonols**					
**3**	2.806	C_33_H_40_O_21_	771.1972	609.1447, 463.0854, 301.0349, 178.9999, 151.0043	Quercetin-3-*O*-rutinoside-7-*O*-glucoside
**4**	2.865	C_27_H_30_O_17_	625.1401	463.0851, 301.0346, 299.0196, 178.9962, 151.0035	Quercetin-3,7-*O*-diglucoside
**5**	3.221	C_33_H_40_O_21_	771.1958	625.1390, 447.0879, 301.0337, 300.0259, 271.0252, 151.0034	Quercetin-3-*O*-sophoroside-7-*O*-rhamnoside
**6**	4.118	C_33_H_40_O_20_	755.2014	609.1433, 285.0393, 431.0911, 255.0302	Kaempferol-3-*O*-sophoroside-7-*O*-rhamnoside
**8**	4.473	C_28_H_32_O_17_	639.1553	477.1011, 315.0500, 301.0300, 300.0262, 271.0249	Isorhamnetin-3,7-*O*-diglucoside
**9**	4.499	C_34_H_42_O_21_	785.2126	623.1602, 477.0988, 315.0507, 300.0267, 151.0052	Isorhamnetin-3-*O*-rutinoside-7-*O*-glucoside
**10**	4.845	C_34_H_42_O_21_	785.2111	639.1535, 461.1051, 315.0500, 300.0283, 151.0039	Isorhamnetin-3-*O*-sophoroside-7-*O*-rhamnoside
**11**	7.443	C_27_H_30_O_16_	609.1443	447.0865, 301.0344, 300.0233, 299.0186	Quercetin-3-*O*-glucoside-7-*O*-rhamnoside
**12**	7.597	C_33_H_40_O_20_	755.2022	447.0865, 301.0344, 300.0233, 271.0248, 151.0039	Quercetin-3-*O*-rutinoside-7-*O*-rhamnoside
**13**	7.604	C_27_H_30_O_17_	625.1401	301.0302, 300.0271, 271.0239, 255.0305, 151.0032	Quercetin-3-*O*-sophoroside
**14**	8.069	C_27_H_30_O_17_	625.1401	317.0271, 287.0191, 271.0237, 151.0034, 125.0231	Myricetin-3-*O*-rutinoside
**15**	8.230	C_33_H_40_O_20_	755.2022	593.1499, 447.0921, 285.0376, 271.0198	Kaempferol-3-*O*-rutinoside-7-*O*-glucoside
**16**	8.493	C_44_H_50_O_25_	977.2535	832.2069, 771.1926, 625.1458, 545.0402, 447.0858, 301.0302, 299.0186	Quercetin-3-*O*-(-6-*O*-sinapoyl)-sophoroside-7-*O*-rhamnoside
**17**	8.603	C_21_H_20_O_13_	479.0819	317.0246, 316.0197, 287.0184, 271.0246, 214.0274	Myricetin-3-*O*-galactoside
**18**	8.802	C_43_H_48_O_24_	947.2414	801.1856, 771.2014, 625.1439, 447.0885, 301.0365, 271.0212	Quercetin-3-*O*-sophoroside-O-glucuronide-7-*O*-rhamnoside
**19**	9.017	C_33_H_40_O_19_	739.2073	593.1483, 285.0378, 284.0321, 125.0245	Kaempferol-3-*O*-rutinoside-7-*O*-rhamnoside
**20**	9.068	C_28_H_32_O_16_	623.1603	477.0993, 461.1008, 315.0495, 285.0375, 275.0158	Isorhamnetin-glucoside-rhamnoside
**21**	9.136	C_27_H_30_O_15_	593.1511	447.0914, 431.0927, 285.0400, 255.0299	Kaempferol-3-*O*-glucoside-7-*O*-rhamnoside
**22**	9.212	C_34_H_42_O_20_	769.2179	623.1594, 315.0474, 299.011, 285.0354	Isorhamnetin-3-*O*-rutinoside-7-*O*-rhamnoside
**23**	9.254	C_27_H_30_O_16_	609.1443	429.0860, 285.0367, 255.0325, 227.0374, 151.0038	Kaempferol-3-*O*-sophoroside
**24**	9.423	C_34_H_42_O_20_	769.2179	315.0490, 299.0187	7-Methylquercetin-3-galactoside-6″-rhamnoside-3‴-rhamnoside
**25**	9.559	C_45_H_52_O_25_	991.2686	845.2133, 639.1541, 477.1049, 315.0484, 300.0202, 275.0174	Isorhamnetin-3-*O*-(-6-*O*-sinapoyl)-sophoroside-7-*O*-rhamnoside
**26**	9.601	C_28_H_32_O_16_	623.1598	477.1057, 461.1088, 315.0502, 271.0226, 183.0470	Isorhamnetin 3-*O*-glucoside-7-*O*-rhamnoside
**27**	10.016	C_43_H_48_O_23_	931.2484	785.1874, 609.1463, 465.0655, 285.0388, 125.0253	Kaempferol-3-*O*-sophoroside-*O*-glucuronide-7-*O*-rhamnoside
**28**	10.024	C_44_H_50_O_24_	961.2598	815.2039, 639.1500, 461.1050, 485.01212, 315.0480, 300.0268, 135.0301	Isorhamnetin-3-*O*-sophoroside-*O*-glucuronide-7-*O*-rhamnoside
**29**	10.253	C_27_H_30_O_15_	593.1500	461.1059, 447.0907, 315.0506, 300.0260, 285.0360, 270.0138	Isorhamnetin-3-*O*-arabinoside-7-*O*-rhamnoside
**30**	10.295	C_27_H_30_O_16_	609.1441	301.034, 300.0274, 271.0250, 178.9991, 151.0035	Quercetin-3-*O*-rutinoside
**31**	10.870	C_27_H_30_O_15_	593.1512	447.0885, 315.0537, 300.0289, 285.0412, 227.0363	Kaempferol-3-*O*-galactoside-7-*O*-rhamnoside
**32**	10.956	C_28_H_32_O_17_	639.1553	331.0440, 330.0378, 317.0239, 315.0140, 287.0204, 271.0237, 215.0364, 178.9997, 151.0032	Myricetin-3′-methyl-3-*O*-rutinoside
**33**	11.051	C_21_H_20_O_12_	463.0876	301.0299, 272.0293, 255.0310, 217.0233, 151.0035	Quercetin-3-*O*-galactoside
**34**	11.522	C_21_H_20_O_12_	463.0876	301.0324, 271.0248, 255.0299, 227.0355, 151.0042	Quercetin-3-*O*-glucoside
**35**	13.519	C_27_H_30_O_15_	593.1495	285.0394, 255.0296, 227.0355	Kaempferol-3-*O*-rutinoside
**36**	14.314	C_28_H_32_O_16_	623.1598	315.0502, 301.0308, 285.0406, 271.0252, 255.0290	Isorhamnetin-3-*O*-rutinoside
**38**	14.957	C_21_H_20_O_11_	447.0918	285.0380, 255.0290, 227.0347	Kaempferol-3-*O*-glucoside
**39**	15.550	C_23_H_24_O_13_	507.1131	344.0537, 301.0355, 287.0582, 273.0399, 258.0168	Syringetin-3-*O*-galactoside
**40**	15.609	C_22_H_22_O_12_	477.1024	315.0502, 300.0266, 285.0392, 271.0251, 151.0042	Isorhamnetin-3-*O*-glucoside
**41**	16.278	C_22_H_22_O_12_	477.1028	315.0503, 300.0208, 285.0241, 299.0242	Isorhamnetin-3-*O*-galactoside
**42**	17.466	C_15_H_10_O_8_	317.0296	178.09979, 151.0038, 137.0243, 107.0138, 83.0145	Myricetin
**44**	18.63	C_21_H_20_O_11_	447.0921	301.034, 300.0262, 151.0029, 121.0295, 107.0133	Quercetin-7-*O*-rhamnoside
**48**	20.672	C_43_H_54_O_22_	921.2998	759.2463, 722.0562, 447.0300, 286.0375, 285.0362	3-*O*-β-D-sophorosyl-kaempferol-7-*O*-{3-*O*-[2(*E*)-2,6-dimethyl-6-hydroxy-2,7-octadienoyl]}-α-L-rhamnoside
**50**	22.049	C_21_H_20_O_10_	431.0980	344.0179, 285.0403, 284.0321, 257.0436, 254.9958	Kaempferol-rhamnoside
**51**	22.442	C_27_H_30_O_15_	593.1291	487.0871, 447.0906, 285.0390, 284.0328, 255.0297, 151.0014	Kaempferol-3-*O*-glucoside-7-*O*-rhamnoside
**52**	22.624	C_22_H_22_O_11_	461.1089	431.0113, 315.0536, 299.0167, 287.0528, 271.0239, 107.0142	Isorhamnetin-rhamnoside
**53**	22.692	C_15_H_10_O_7_	301.0350	178.9981, 163.0049, 151.0039, 107.0139, 65.0032	Quercetin
**55**	25.436	C_37_H_44_O_17_	759.2483	597.2049, 596.1804, 447.0926, 358.8980, 285.0427	3-*O*-β-D-glucosyl-kaempferol-7-*O*-{2-*O*-[2(*E*)-2,6-dimethyl-6-hydroxy-2,7-octadienoyl]}-α-L-rhamnoside
**59**	28.803	C_15_H_10_O_6_	285.0399	241.0512, 229.0497, 154.0426, 107.0141, 93.0356, 63.0365	Kaempferol
**60**	28.201	C_16_H_12_O_7_	315.0503	301.0300, 300.0272, 271.0249, 227.0398, 151.0044, 107.0134	Isorhamnetin
**New Flavonoids**					
**43**	17.64	C_43_H_54_O_23_	937.2957	773.8043, 629.1837, 609.1457, 457.7284, 447.1069, 301.0346,	Quercetin-3-*O*-rutinoside-7-*O*-[2(*E*)-2,6-dimethyl-6-hydroxy-2,7-octadienoyl(1→6)]-glucoside
**45**	19.129	C_44_H_56_O_23_	951.3107	767.2035, 623.1589, 459.0922, 315.0515, 313.0312	Isorhamnetin-3-*O*-rutinoside-7-*O*-[2(*E*)-2,6-dimethyl-6-hydroxy-2,7-octadienoyl(1→6)]-glucoside
**46**	19.519	C_38_H_46_O_19_	805.2537	643.1941, 477.1037, 458.5028, 315.0494, 313.0336, 299.0067,	Isorhamnetin-3-*O*-glucoside-7-*O*-[2(*E*)-2,6-dimethyl-6-hydroxy-2,7-octadienoyl(1→6)]-glucoside
**47**	19.578	C_43_H_54_O_23_	937.2957	775.2385, 668.9043, 609.1446, 463.0873, 447.0838, 301.0346,	Quercetin-3-*O*-sophoroside-7-*O*-[2(*E*)-2,6-dimethyl-6-hydroxy-2,7-octadienoyl(1→2)]-rhamnoside
**49**	20.911	C_44_H_56_O_23_	951.3107	789.2574, 623.1507, 477.1026, 315.0499, 299.0190	Isorhamnetin-3-*O*-sophoroside-7-*O*-[2(*E*)-2,6-dimethyl-6-hydroxy-2,7-octadienoyl(1→2)]-rhamnoside
**56**	25.552	C_38_H_46_O_18_	789.2582	627.2021, 477.0975, 315.0502, 300.0324, 299.0218, 286.0425	Isorhamnetin-3-*O*-glucoside-7-*O*-[2(*E*)-2,6-dimethyl-6-hydroxy-2,7-octadienoyl(1→2)]-rhamnoside
**Flavanols**					
**1**	2.595	C_15_H_14_O_7_	305.0667	219.0669, 164.0113, 139.0417, 125.0251	Gallocatechin
**2**	2.647	C_15_H_14_O_7_	305.0667	219.0658, 164.0116, 139.0404, 125.0250	Epigallocatechin
**7**	4.348	C_15_H_14_O_6_	289.0715	245.0813, 219.0279, 203.0713, 125.0239, 109.0298	Epicatechin
**Flavane**					
**58**	25.863	C_15_H_12_O_5_	271.0606	151.0035, 119.0497, 107.0144, 83.0138, 63.0246	Naringenin
**Unknown**					
**37**	14.907	C_43_H_48_O_24_	947.2414	783.6926, 639.1375, 609.1426, 486.9109, 301.0346, 285.3524,	Unidentified
**54**	23.800	C_37_H_44_O_18_	775.244	612.1838, 510.0540, 463.0849, 301.0333, 299.0196	Unidentified
**57**	25.713	C_38_H_46_O_18_	789.2582	627.1983, 477.1037, 315.0500, 299.0164	Unidentified

## Data Availability

Data is contained within the article and Supplementary Materials.

## Supplementary Materials

The following supporting information can be downloaded at: https://www.mdpi.com/article/10.3390/antiox13070763/s1, Figure S1: Total ion current chromatography of FCL in negative ion mode; Figure S2: Total ion current chromatography of mixed standards in negative ion mode; Figure S3: Possible fragmentation pathway of isorhamnetin-3-*O*-rutinoside-7-*O*-glucoside in MS/MS; Figure S4: Possible fragmentation pathways of 6 new compounds; Figure S5: UPLC-DAD chromatogram of flavonoids in FCL; Table S1: Content of 16 flavonoids in Chinese sea buckthorn leaves/(mg·g^−1^ DW).
