# Supplementary material for: Antioxidant and Stress Resistance Properties of Flavonoids from Chinese Sea Buckthorn Leaves from the Qinghai–Tibet Plateau
Source: Antioxidants (Basel). 2024 Jun 25;13(7):763. doi: 10.3390/antiox13070763 (PMC11273727; doi:10.3390/antiox13070763)
Supplement: Supplementary file 1 [file antioxidants-13-00763-s001.zip › antioxidants-3035474-supplementary.pdf]

Supplementary Data

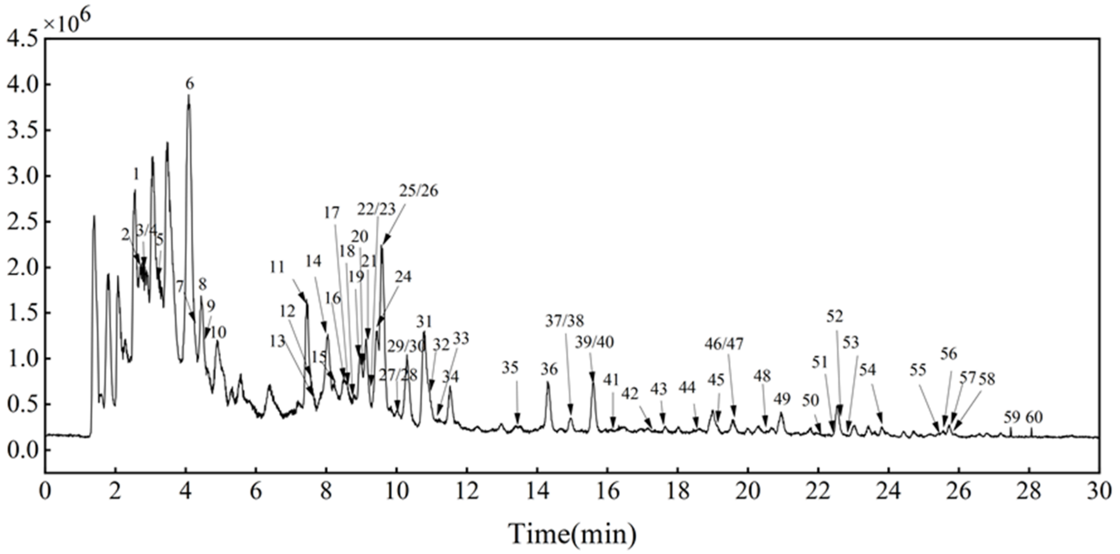

Figure S1. Total ion current chromatography of FCL in negative ion mode.

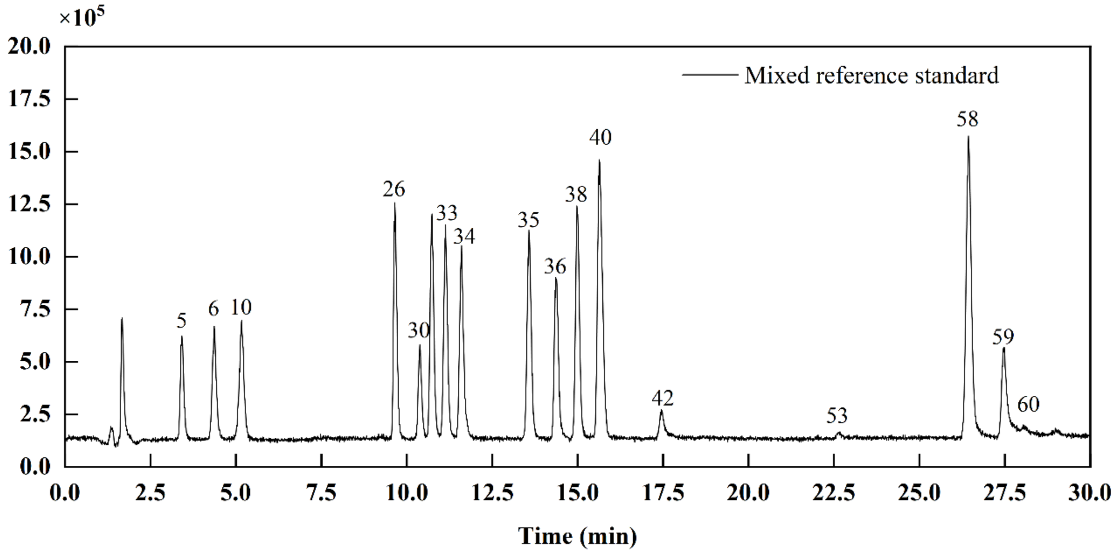

Figure S2. Total ion current chromatography of mixed standards in negative ion mode.

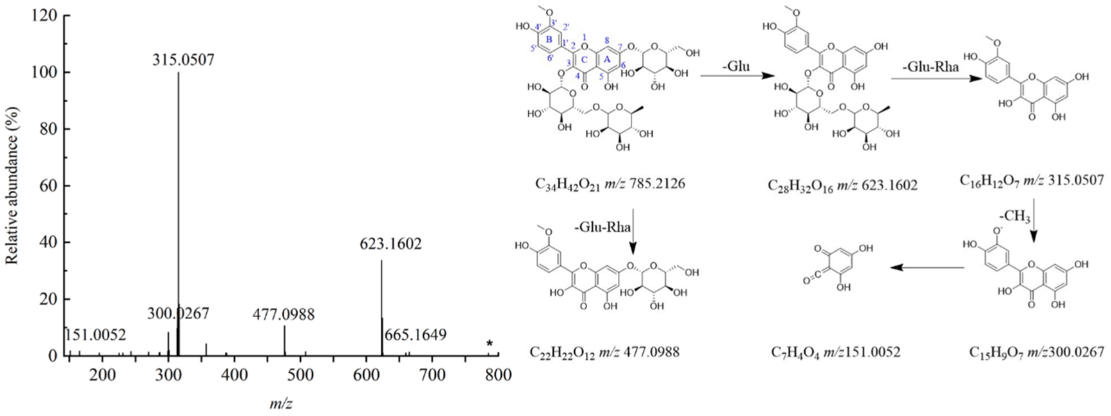

Figure S3. Possible fragmentation pathway of isorhamnetin-3-*O*-rutinoside-7-*O*-glucoside in MS/MS.

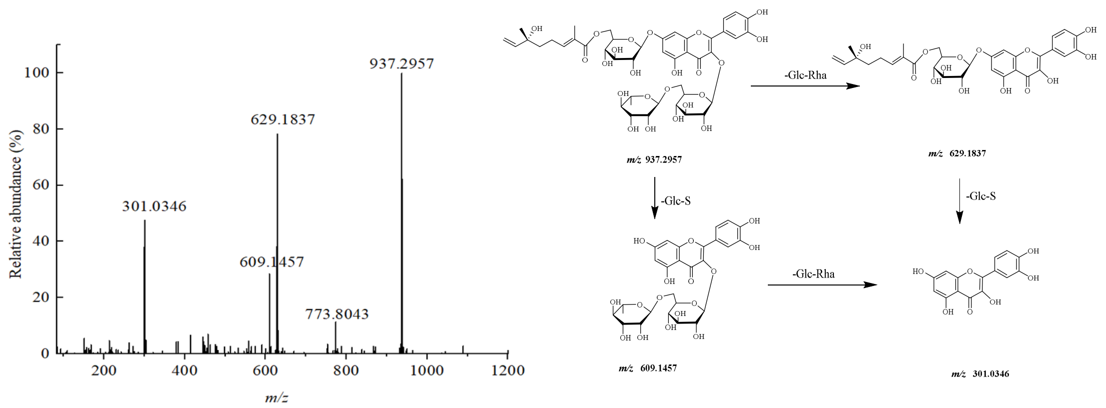

Compound **43**: Quercetin-3-*O*-rutinoside-7-*O*-[2(*E*)-2,6-dimethyl-6-hydroxy-2,7-octadienoyl(1→6)]-glucoside

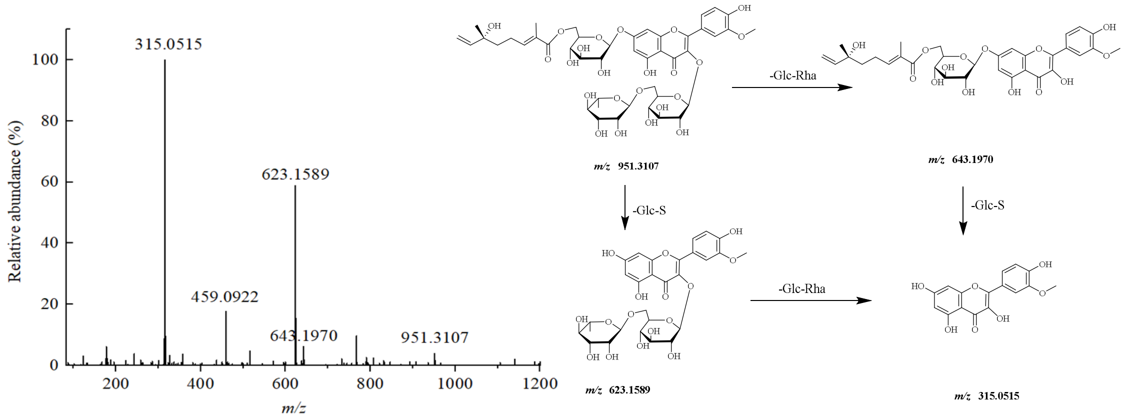

Compound **45**: Isorhamnetin-3-*O*-rutinoside-7-*O*-[2(*E*)-2,6-dimethyl-6-hydroxy-2,7-octadienoyl(1→6)]-glucoside

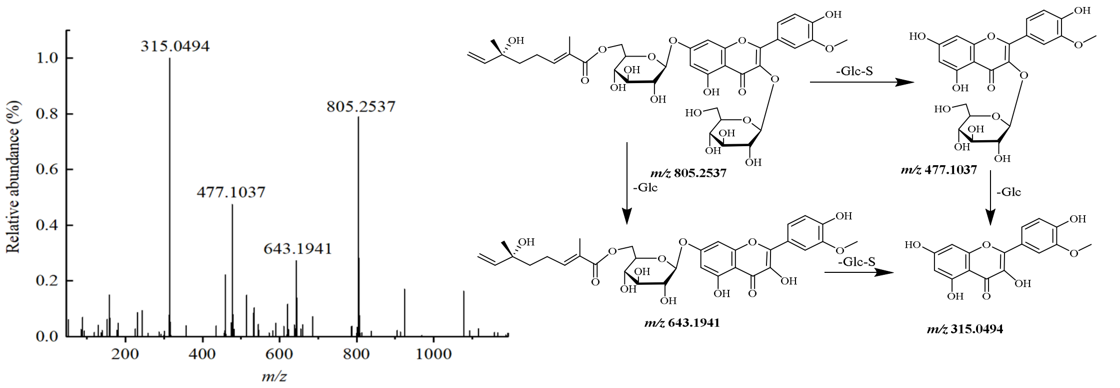

Compound **46**: Isorhamnetin-3-*O*-glucoside-7-*O*-[2(*E*)-2,6-dimethyl-6-hydroxy-2,7-octadienoyl(1→6)]-glucoside

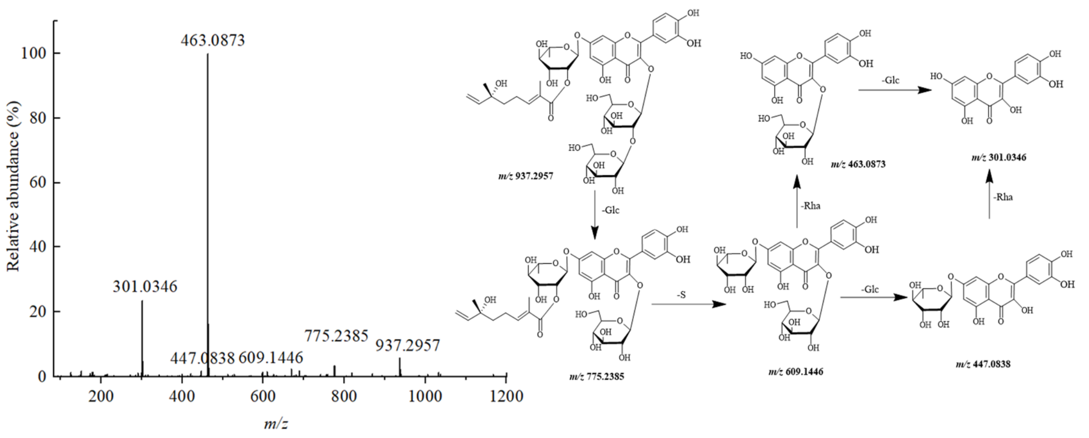

Compound **47**: Quercetin-3-*O*-sophoroside-7-*O*-[2(*E*)-2,6-dimethyl-6-hydroxy-2,7-octadienoyl(1→2)]-rhamnoside

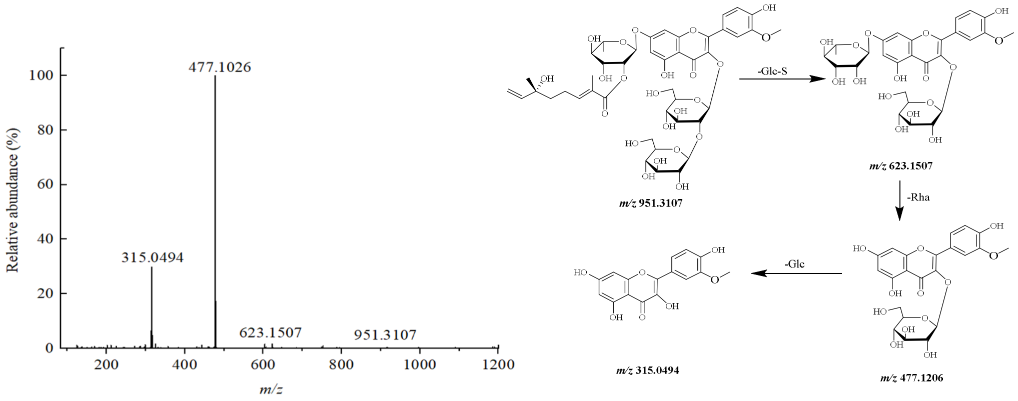

Compound **49**: Isorhamnetin-3-*O*-sophoroside-7-*O*-[2(*E*)-2,6-dimethyl-6-hydroxy-2,7-octadienoyl(1→2)]-rhamnoside

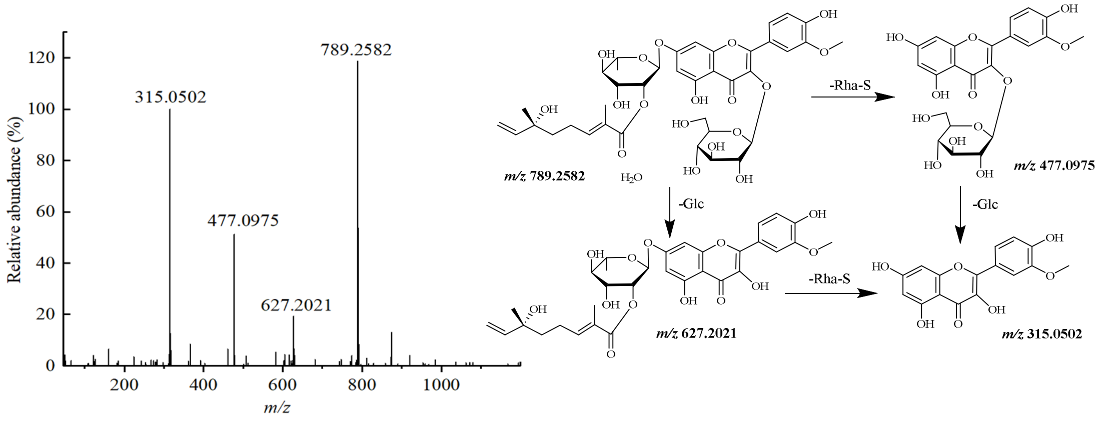

Compound **56**: Isorhamnetin-3-*O*-glucoside-7-*O*- [2(*E*)-2,6-dimethyl-6-hydroxy-2,7-octadienoyl(1→2)]-rhamnoside

Figure S4. Possible fragmentation pathways of 6 new compounds.

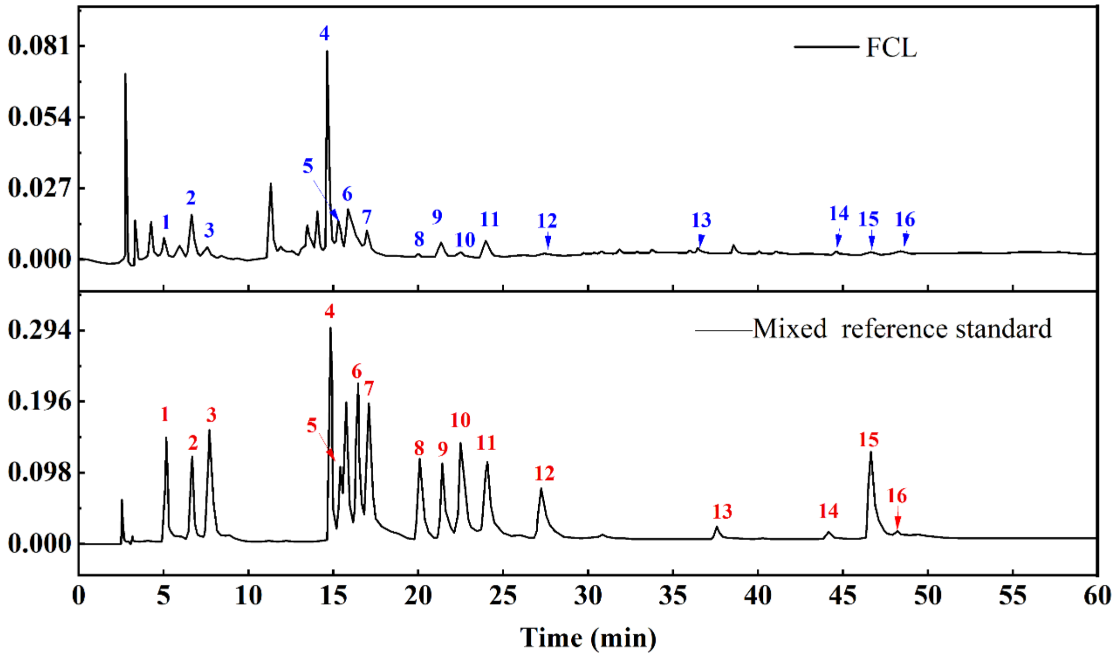

Figure S5. UPLC-DAD chromatogram of flavonoids in FCL.

Table S1. Content of 16 flavonoids in Chinese sea buckthorn leaves/(mg·g^{-1}DW).

Category	Peak	flavonoids	Rt/Min	Contents / (mg·g^{-1}DW)	Total / (mg·g^{-1}DW)
Flavonoid disaccharide chain glycosides	1	Quercetin-3-*O*-sophoroside-7-*O*-rhamnoside	5.142	5.34 ±0.21	28.60±0.35[a]
	2	Kaempferol-3-*O*-sophoroside-7-*O*-rhamnoside	6.686	8.61 ±0.17	
	3	Isorhamnetin 3-*O*-sophoroside-7-*O*-rhamnoside	7.745	3.23 ±0.06	
	4	Isorhamnetin-3-*O*-glucoside-7-*O*-rhamnoside	14.816	11.41 ±0.11	
Flavonoid monosaccharide chain glycoside	5	Quercetin 3-*O*-rutinoside	15.498	8.41 ±0.14	26.88±0.71[b]
	6	Quercetin-3-*O*-galactoside	16.53	8.20±0.22	
	7	Quercetin-3-*O*-glucoside	17.199	3.00 ±0.16	
	8	Kaempferol-3-*O*-rutinoside	20.234	0.33 ±0.00	
	9	Isorhamnetin 3-*O*-rutinoside	21.565	2.40 ±0.18	
	10	Kaempferol-3-*O*-glucoside	22.719	0.81 ±0.11	
	11	Isorhamnetin-3-*O*-glucoside	24.208	2.70±0. 07	
Flavonoid aglycones	12	Myricetin	27.502	1.81 ±0.03	8.87±0.50[c]
	13	Quercetin	36.794	1.98 ±0.05	
	14	Naringenin	44.438	0.22 ±0.00	
	15	Kaempferol	46.936	1.26 ±0.03	
	16	Isorhamnetin	48.434	3.60 ±0.50	
